# Health promotion in schools: a multi-method evaluation of an Australian School Youth Health Nurse Program

**DOI:** 10.1186/s12912-015-0071-0

**Published:** 2015-04-22

**Authors:** Michelle Banfield, Kelly McGorm, Ginny Sargent

**Affiliations:** Australian Primary Health Care Research Institute and National Institute for Mental Health Research, The Australian National University, 63 Eggleston Rd, Acton, ACT 2601 Australia; Program Support Nurse Women Youth and Children Community Health Programs, ACT Health, Level 3, 1 Moore Street, Civic, ACT 2601 Australia; National Centre for Epidemiology and Population Health, The Australian National University, 62 Eggleston Rd, Acton, ACT 2601 Australia

**Keywords:** School nursing, Health promotion, Youth health, Health service access

## Abstract

**Background:**

Health promotion provides a key opportunity to empower young people to make informed choices regarding key health-related behaviours such as tobacco and alcohol use, sexual practices, dietary choices and physical activity. This paper describes the evaluation of a pilot School Youth Health Nurse (SYHN) Program, which aims to integrate a Registered Nurse into school communities to deliver health promotion through group education and individual sessions.

**Methods:**

The evaluation was guided by the RE-AIM (reach, effectiveness, adoption, implementation, maintenance) framework. The objectives were to explore: 1) whether the Program was accessible to the high school students; 2) the impacts of the Program on key stakeholders; 3) which factors affected adoption of the Program; 4) whether implementation was consistent with the Program intent; and 5) the long-term sustainability of the Program. Research included retrospective analysis of Program records, administration of a survey of student experiences and interviews with 38 stakeholders.

**Results:**

This evaluation provided evidence that the SYHN Program is reaching students in need, is effective, has been adopted successfully in schools, is being implemented as intended and could be maintained with sustained funding. The nurses deliver an accessible and acceptable primary health care service, focused on health promotion, prevention and early intervention. After some initial uncertainty about the scope and nature of the role, the nurses are a respected source of health information in the schools, consulted on curriculum development and contributing to whole-of-school health activities.

**Conclusions:**

Findings demonstrate that the SYHN model is feasible and acceptable to the students and schools involved in the pilot. The Program provides health promotion and accessible primary health care in the school setting, consistent with the Health Promoting Schools framework.

**Electronic supplementary material:**

The online version of this article (doi:10.1186/s12912-015-0071-0) contains supplementary material, which is available to authorized users.

## Background

The Health Promoting Schools framework is part of the World Health Organization (WHO) Global School Health Initiative and has the goal of strengthening health promotion and education activities at every level from local to global [1]. The components for delivering a Health Promoting School are: health policies, physical environment, social environment, community involvement, curriculum to develop health skills and the provision of health services at the school [2].

In many countries, health promotion and prevention activities in schools are the role of the school nurse [3,4]. As a result, school nursing has evolved into a very different role from that of traditional first aid provider [5,6]. This is particularly evident in secondary schools where health promotion activities target key health-related behaviours that are established in adolescence such as tobacco and alcohol use, sexual practices, dietary choices and physical activity [7].

In Australia, a school nursing model based on the Health Promoting Schools framework was developed and implemented in Queensland (the School-Based Youth Health Nurse Program in 1999) and Victoria (the Secondary School Nursing Program in 2000). A defining principle of this model is the integration of the nurse into the school community [5]. The focus of the program is to work with school community to promote health and wellbeing for young people in the school setting. The specific goals include providing both a confidential health service for young people including referral to other services as required and health promotion for students, teachers and the wider school community, but exclude the provision of first aid [8,9].

### School Youth Health Nurse Program

In 2009, the Australian Capital Territory (ACT) Government developed a School Youth Health Nurse (SYHN) Program based on the Queensland model and commenced a pilot in eight government high schools (grades 7–10). The Program planning and implementation was guided by a Memorandum of Understanding (MOU) between the health and education departments. The MOU described the SYHN Program and the cross-sector collaboration required, overseen by a reference group consisting of school principals and representatives from ACT Government.

The SYHN Program team consists of Registered Nurses (RNs), with experience in youth health, who deliver the Program within schools, a Clinical Nurse Consultant who provides procedural and clinical supervision, and a Program Manager within the health department. The Program team works with school principals, student welfare team members and senior members of the education department to tailor the Program according to school needs. The nurses work with teachers to assist in the delivery of the health curriculum in class and whole of school forums. The nurses also co-ordinate smaller sessions tailored to student population needs such as smoking cessation and healthy eating groups. The balance of their time is spent in individual consultations with students. Each of the participating schools provides private office space for the SYHN where students may drop-in or attend consultations at pre-appointed times. Each nurse covers two schools, spending two days per week in each school and one day in the central office for team meetings, debriefing, organising referrals, planning health promotion activities, and staff development.

In 2012, the authors were asked to evaluate the pilot SYHN Program. The objectives of this study, guided by the RE-AIM framework [10,11] were to explore: 1) whether the Program was accessible to the high school students (reach); 2) the impacts of the Program on key stakeholders (effectiveness); 3) factors that affected adoption of the Program (adoption); 4) whether implementation was consistent with the Program model (implementation); and 5) long-term sustainability of the Program (maintenance).

## Methods

A retrospective evaluation was conducted by the authors and guided by an advisory group consisting of government and school stakeholders. The research had approval from The Australian National University Science and Medical Delegated Ethics Review Committee, the ACT Health Human Research Ethics Committee (HREC), the ACT Health HREC Survey Resource and Approval Sub-committee, the ACT Education and Training Directorate Planning and Performance Branch and the school principals. All interview participants gave written consent; student participation required both student and parent written consent.

### Evaluation framework

The evaluation was guided by the RE-AIM framework [10,11]. This framework was designed to assess complex health promotion interventions in “real world” settings and examine the reach, effectiveness, adoption, implementation and maintenance of a program [11]. The RE-AIM dimensions and their relationship to the research questions and data sources are presented in Table 1.

**Table 1:** **Study application of the RE-AIM dimensions** [10,11]

**Dimension**	**Key indicators (from Glasgow et al.)**	**Current study focus areas**	**Key data sources**
Reach	• Percent of target population	• Knowledge about the program within schools	• Program documents
	• Representativeness (those in need/left out)	• Characteristics of students accessing program (e.g. gender, age)	• Program data
		• Barriers & enablers	• Student survey
			• Interviews with nurses and school staff
Effectiveness	• Impact on key outcomes (satisfaction, quality of life)	• Student satisfaction	• Student survey
	• Unanticipated negative outcomes	• Impact on schools	• Interviews with nurses, school staff and external services
		• Impact on external services	
Adoption	• Percent of settings participating in program	• Barriers & enablers	• Program data
	• Feasibility for all settings including low resource	• Effects of socio-economic disadvantage	• Student survey
			• Interviews with nurses, school staff and external services
Implementation	• Consistency of program delivery	• Delivery of program according to guidelines	• Program documents
	• Costs	• Challenges	• Interviews with nurses and government stakeholders
Maintenance	• Long-term improvements for individuals	• Nurse satisfaction	• Program documents
	• Long-term modifications/sustainability for settings	• Long-term effects on schools	• Interviews with nurses, school staff and government stakeholders
		• Factors affecting sustainability	• Nurse survey

### Data sources

#### Program documentation

The MOU, Program guidelines and meeting notes were used to compile information regarding Program aims and implementation. De-identified Program activity records kept by each nurse for 2010 and 2011 were used to examine the following characteristics of contacts with students: number of contacts; nature of health concern; student gender; Aboriginal or Torres Strait Islander (ATSI) or cultural and linguistically diverse (CALD) background; student grade (7–10); mode of delivery (individual or group); and external service referrals.

To complement these data, qualitative and quantitative data were collected by the authors from August-October 2012: interviews with Program stakeholders and surveys of nurses, students and parents. To explore students’ knowledge and experience of the SYHN Program, a **student survey** was developed from existing sources [3,12] and adapted for the local context. The survey asked closed ended questions in the following areas:

- whether students knew about the nurse and if so by what means;
- if they had attended an appointment with the nurse;
- barriers and enablers for accessing the nurse [adapted from 3]
- their perception of the activities delivered by the nurse [adapted from 3]
- if applicable, their satisfaction with care received.

The satisfaction scale and barriers/enablers section comprised Likert-type scales and yes/no responses. An online survey asking the same questions was available for parents to complete.

Face-to-face, in-depth **interviews** with program stakeholders, school staff and nurses were conducted according to a semi-structured protocol, with a topic guide covering each domain of the RE-AIM framework (see Additional file 1 for indicative questions). A **nurse survey**, to supplement the nurse interviews, consisted of a short, paper-based questionnaire to measure work satisfaction [13], tasks [3] and professional development opportunities [14].

### Participants and procedure

#### School Youth Health Nurses

The four RNs who had implemented the Program since its commencement in 2009 were invited to participate in individual interviews about their experiences.

#### Schools: staff, students and parents

The eight schools participating in the SYHN Program were geographically dispersed across the ACT and the socio-demographic characteristics of their surrounding areas varied. The Program targeted students in Grades 7–10, the conventional high school grades in the ACT. However, some schools in the pilot extended beyond these grades, resulting in a small number of contacts with students outside the target group. A total of approximately 4100 students were enrolled in Grades 7–10 (range 200–700 students per school) in 2011. Each of these schools participated in this evaluation to varying degrees according to school principal preferences.

Staff interviews were conducted for all eight schools. The principal or deputy principal and a member of teaching or welfare staff within each school were individually interviewed.

Principals from six schools facilitated the conduct of the student survey. The principals at the remaining two schools did not feel they had the capacity to do so. Information and consent forms were distributed by school staff to the entire student body of five schools, and to selected classes in one school with limited capacity. Parents were invited to take part in an online questionnaire via the information sheet accompanying the student consent form.

#### Other Program stakeholders

Government employees involved in the SYHN Program were invited to participate in a semi-structured interview regarding their knowledge of, and experiences with, its implementation. Additionally, eight services to which the nurses referred students were identified. These included youth and Aboriginal health services, sexual health centres, mental health and child protection services. Service personnel who had received referrals from the nurses were invited to participate in a semi-structured interview regarding their experiences of the Program.

### Analysis

Data were analysed at the Program level in order to protect the identity of the schools, nurses and students. Quantitative data were analysed using Statistical Package for the Social Sciences 20 (SPSS 20, IBM). Primary analyses consisted of descriptive statistics, including percentages and total scale scores [13].

All qualitative data, including Program documents and interview transcripts were collated in NVivo 9 (QSR International). A thematic analysis was conducted using the RE-AIM domains as an *a priori* coding framework. Transcripts and documents were analysed using a combination of deductive and inductive coding. Sources were first coded to the broad RE-AIM dimensions, with sub-themes developed inductively from the data in the course of coding. Results are presented according to these dimensions with emerging themes discussed.

## Results

A total of 38 interviews were conducted: the four nurses; 17 school staff, representing all schools; eight stakeholders from seven external services; and nine government stakeholders from the health, education and community services departments. A total of 290 students from six schools completed the student questionnaire. Response to the parent survey was very low (seven respondents) and insufficient for analysis.

### Reach of individual health service

The Program data consisted of a total of 3670 recorded contacts with nurses across the two year period, including both individual and group contacts. Table 2 summarises the characteristics of students accessing the Program. Group sessions are counted as only one contact; however, the total number of students attending group sessions is included. As the records were anonymous, students who accessed the SYHN several times in a year individually or in group sessions will be recorded as multiple contacts.

**Table 2:** **Characteristics of students accessing the program as recorded in program data**

		**2010 (N = 1907)**		**2011 (N = 1763)**	
**Characteristic**		**n**	**%**	**n**	**%**
Demographics	Male	493	25.8	473	26.8
	Female	1402	73.5	1430	81.1
	ATSI	123	6.5	134	7.6
	CALD	59	3.1	55	3.1
School grade	Grade 6	22	1.2	22	1.2
	Grade 7	202	10.6	199	11.3
	Grade 8	340	17.8	389	22.1
	Grade 9	670	35.1	402	22.8
	Grade 10	399	20.9	556	31.5
	Mixed grades	26	1.4	88	5.0
Groups		305	16	309	17.5
	Size	2 to 680		2 to 450	
	Total participants	5006		5926	
Health area	Mental health	1034	54.2	954	54.1
	General health	404	21.2	494	28
	Sexual health	335	17.6	284	16.1
	Drug and Alcohol	242	12.7	290	16.4

These data indicate Program uptake varied between year and grades. In 2010 the fewest contacts were with Grade 7 students (10.6%, n = 202) and the greatest with Grade 9 students (35.1%, n = 670). In 2011, contacts ranged from 11.3% (n = 199) with Grade 7 students to 31.5% (n = 556) with Grade 10 students. This is consistent with the observations from nurses and school staff of a higher level of risk taking behaviours in Grade 9 and 10 students (particularly experimentation with alcohol, other drugs and sexual behaviour). These students were also accessing the Program more than other grades.

*We’ve got a small but significant population of kids that get involved in some pretty hard core risk taking …. [The nurse is] quite good at building a rapport with them, and she becomes trusted by them, and they will readily seek her out and get that support…and the referral that they need.* School staff member

Program data indicate that only 25.8% (n = 493) to 26.8% (n = 473) of contacts involved boys. The nurses commented that boys are a hard group to reach, particularly for individual consultations.

**Figure 1:**
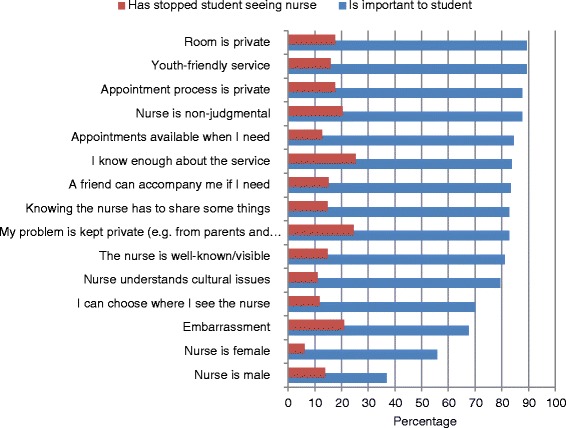
Factors affecting students accessing the SYHN. The data series “is important to student” represents the percentage of survey respondents who reported each factor as important when they were thinking about accessing the nurse. The data series “has stopped student seeing the nurse” represents the percentage of students who reported that the factor had actually stopped them accessing the nurse.

Figure 1 presents the percentage of student survey respondents who endorsed factors that had stopped them seeing the nurse and the percentage of respondents who reported these factors were “somewhat” or “very” important when they considered accessing the nurse. Factors that stopped students accessing the service were lack of knowledge, privacy concerns and embarrassment. The factors students reported as most important to them when they considered accessing the nurse were privacy, and that the nurse was non-judgmental and youth friendly. School staff noted that students felt there was a stigma associated with attending an appointment with school counsellors, but that this stigma was not associated with seeing the nurse.

*I’m surprised at how few barriers there are…My main experience is getting kids to see the counsellor and that is quite difficult, but kids who need [the nurse] tend to seek her out.* School staff member

The nurses and some school staff named the location of the nurse’s office as a key factor in reaching students. They felt that locating the office in a main student area, usually away from disciplinary staff, increased the nurse’s visibility and ease of access. However, school staff preferred the nurse to be located with the student welfare team, and these were not always in a main student area.

*To get her integrated you need to find the ideal office space. That was one of the things we struggled with in the beginning. [The nurse] felt isolated and she wasn’t being accessed. And we actually, since she’s been here, have moved her office a couple of times. And we’ve found the best place is right in the heart of where the kids are.* School staff member

### Reach of group health education

The four nurses recorded total attendance at group health education of 5006 students in 2010 and 5976 students in 2011 (total school enrolments were approximately 4100 each year). This included both small group sessions and contributions to curriculum in class settings. Interviews with nurses and school staff indicate that schools employed a variety of methods to promote the Program to students and staff and to maximise the chances of reaching the students most in need. Seventy-eight percent (n = 226) of the student survey respondents reported that they are aware that their school had a SYHN. Students most commonly learned about the nurse from teachers (45%, n = 130), through talks and presentations (38%, n = 110) or from other students (27%, n = 79).

School staff identified the nurses working only two days per week in each school as an important barrier to the Program achieving its potential reach. School staff thought that if nurses were in schools full time, more time could be spent in small group, classroom and whole-of-school activities.

*I think she mainly concentrates on individual consultations. Only being here for two days a week and having a student population of over 700, her time is limited. I’d like to see her working more in classes, working with staff on the delivery of the health curriculum. I think that would be a way forward, but that’s a matter of balance really…* School staff member

### Effectiveness - What are the reported impacts of the Program?

#### Impact on students

Student survey respondents who indicated they had visited the SYHN (15.2% of respondents, n = 44) were asked to complete satisfaction questions (Table 3). The majority of respondents (75%, n = 33) indicated that their experience was good or excellent. When asked about their perception of change in their health as a result of seeing the nurse, 47.7% (n = 21) responded that their health had improved, 50% (n = 22) reported no change.

**Table 3:** **Student satisfaction with the SYHN Program**

**Question**	**Response**	**Number**	**Percentage**
As a result of visiting the nurse, would you say your health and wellbeing is…	Worse	1	2.3
	Same	22	50.0
	Better	21	47.7
How comfortable were you visiting the nurse?	Not at all	3	6.8
	Not very	2	4.5
	Somewhat	22	50.0
	Very	17	38.6
How easy is it to talk to the nurse?	Very hard	2	4.5
	Hard	0	0
	Not very easy	4	9.1
	Easy	25	56.8
	Very easy	13	29.5
How likely are you to follow the nurse’s advice?	Probably not	3	6.8
	Maybe	6	13.6
	Likely	20	45.5
	Very likely	15	34.1
Was the nurse available when you needed?	Rarely	4	9.1
	Some of the time	6	13.6
	Most of the time	21	47.7
	All of the time	13	29.5
Were you included in decision-making about your care?	Yes	23	53.5
	No	6	14.0
	Not sure	14	32.6
Did you feel like your visit was private?	Yes	32	72.7
	No	7	15.9
	Not sure	5	11.4
How would you rate your experience with the nurse overall?	Poor	3	6.8
	Okay	8	18.2
	Good	22	50.0
	Excellent	11	25.0

Nurses, school staff and external stakeholders were unanimous that the SYHN Program had a positive effect on the health and wellbeing of students in the pilot schools. Some suggested the positive effects extended to decreased truancy and improved educational outcomes which may improve longer-term social and economic prospects, particularly for the very high-risk, disengaged students.

*…take for example that group of [Grade] 10 girls, I couldn’t see them being in the position where they are today without that support from the nurse every week…Attendance being improved, health being improved, decision making, behaviour in class, possibly even grades improvements, that’s just from that group.* School Principal

#### Impact on external services

External service providers thought the SYHN Program resulted in an increase in appropriate referrals to their service and appreciated the nurses’ facilitation for school outreach programs. Although often understaffed, external organisations said they could spend more time in schools conducting outreach programs if the nurses had greater availability to assist. However, they also commented that limitations at the policy level on the scope of practice of the nurses reduced the Program’s effectiveness. For example, some participants commented that as trained health professionals, the nurses’ scope of practice should extend to sexual health such as pregnancy testing. Attempts by the nurses to coordinate these activities with external organisations had mixed success due to the need for referral. Nurses were not permitted to transport or accompany students to other services.

### Was the program adopted?

The SYHN Program was successfully adopted in all pilot schools and by the external services. Staff from each school expressed their enthusiasm for the Program and felt the nurses had become an integral part of the student welfare teams.

There were occasional difficulties with role delineation particularly between school counsellors and SYHNs, but as role clarity developed over the first year, these problems tended to settle.

*The nurse has a lot of initiative so she drives things a lot, so, I think the nurse, she knows where she is. She knows her position, what she’s doing, and she knows [her] role, and I’ve even had conversations where she said, ‘Oh, no, we should leave that one to the counsellor’, or ‘Maybe she should go to the youth worker’…* School staff member

Most thought that it had taken 6–12 months for the Program to be fully adopted, requiring active promotion both within and outside the school. After some initial uncertainty about the scope and nature of the role, the nurses became a respected source of health information in the schools, consulted on curriculum development and contributing to whole-of-school health activities.

The importance of good teamwork was seen as central to the successful adoption of the SYHN Program into school communities. Factors reported as assisting good teamwork were: nurse involvement in staff meetings, professional development activities with teachers and administrative staff (such as training on anaphylaxis), and ongoing orientation for new school staff.

### Was the program implemented as planned?

The nurses characterised the service they were providing as “solid and accessible” primary health care, focusing on information, early intervention and referral, in accordance with the SYHN Program model. The government stakeholders attributed this success to a combination of the preparatory work that went into the MOU and Program guidelines and the flexibility of the nurses to adapt to the particular needs of the schools. The Program data revealed that the majority of students accessing the Program were seeking advice on mental health issues or general health issues with the remainder being split between sexual health and drug and alcohol advice (Table 2). The nurses were occasionally performing some “out of scope” tasks such as assisting students who were injured at school, but they felt that it was acceptable that their clinical training was occasionally used to support first aid workers in schools, particularly with serious incidents such as head injuries. Overall, school staff expressed their desire for an increase in the nurses’ involvement in delivering the health curriculum into the future, viewing this as a greater focus on health promotion. However, the nurses took the broader view that everything they did to empower students was health promotion.

*Well I consider what I do actually is health promotion … I suppose they mean more as a whole school health promotion. So for example, we had a number of students that were diabetics and so … I got them together and they formed a bit of a group and we did a thing at assembly on what is diabetes, how do you look after a friend with diabetes and stuff like that, so that kind of whole school thing. But I think every day I do health promotion in the stuff that I do with young people.* School Youth Health Nurse

#### Factors affecting implementation

Time challenges meant some nurses had difficulty achieving a balance between activities. Interviewees also reported nurse participation in scheduled meetings and contribution to curriculum was hindered due to only working two days per week at each school.

*I would see [the school]… needs somebody there full time, you know, four days a week. My dream would be to have two days of consultation only and then one day of small group…and then one day of health promotion…I don’t think that would get on top of the need either, but at least… it would just be better planned, whereas it feels quite chaotic and I always wonder if I’m doing best practice when I’m trying to keep… my head above water. I don’t think that’s ideal.* School Youth Health Nurse

Despite the challenges posed by the limited time the nurses had in schools, school staff members were so enthusiastic about the benefits of the SYHN Program, they were willing to continue with a part-time nurse if it meant that other schools were given the opportunity to have this valued resource.

The nurses found it difficult to provide a private, youth friendly service if they did not have appropriate resources: a dedicated room, reliable access to a phone and computer, and health promotion tools such as posters, brochures and models. These resources were minimum requirements under the Program guidelines but the facilities made available by schools were not always considered ideal by the nurses.

### What factors affect long-term maintenance of the program?

The issues stakeholders considered most important to maintain the integrity of the Program were student confidentiality, maintaining the focus on health promotion, and effective teamwork between nurses and school staff. Issues that required adaptation at the school level were consistency with school policies (e.g., students out of class), the referral between other welfare team members and the needs of teaching staff.

*I think it’s really important for the nurse and the principal to talk about what is and isn’t OK within that school setting. Some schools are more liberal than others…So there’s also that conversation to be held around as what are the expectations of this school and how will the nurse fit into this school, judging on the dynamics.* School Principal

#### Nurse satisfaction

Although there were challenges involved, nurses were highly satisfied with their role. Total scores on the job satisfaction scale [13] ranged from 78 to 94 out of a possible 105, and overall job satisfaction was rated as “very” or “extremely” satisfied. The nurses reported that they had good opportunities to attend training and conferences, but they felt a dedicated course on School Youth Health Nursing would be useful. They had developed a number of Standard Operating Procedures around difficult issues and participated in monthly training sessions on child protection issues. The nurses and the management team emphasised the importance of the weekly office day for debriefing, supervision and peer support.

#### Challenges to sustainability

Although evaluation data from all stakeholder groups indicated that the students value the nurses’ confidentiality policy, some school staff named it as a barrier to effective teamwork. Those in favour of a team approach to student welfare suggested this was not in the best interest of the student, whereas nurses as health professionals are bound by health privacy laws and felt that confidentiality was key to their success with the students.

*There’s a lot of pressure in the schools to provide information about students. They have a different level of confidentiality. So it’s really working out that common good, for what’s best for the student … as well as providing confidential service, because we won’t see anybody if it’s not confidential.* School Youth Health Nurse

Government stakeholders commented that expansion of the SYHN Program beyond the pilot presented a number of potential challenges including office space, team dynamics and ensuring effective and timely integration into new schools.

## Discussion

This evaluation adds considerably to our understanding of school-based nurse-led health promotion programs, with good evidence that the pilot Program is reaching the majority of students in need, is reported as having positive effects on student health and wellbeing, has been adopted successfully in schools and by other youth services, is being implemented as intended, and could be maintained with sustained resourcing. Earlier literature describing nurses’ delivery of a similar health promotion model is limited to description of their roles and responsibilities [5,8,15]; the experiences of the nurses delivering health education [16]; improving access for boys [17]; and a qualitative assessment of whether they are delivering ‘true health promotion’ [18].

A number of key themes emerged across the RE-AIM dimensions and are discussed here.

### Access

It is widely acknowledged that the barriers for young people accessing primary health care are consistent and may be categorized as: acceptability, availability, accessibility, and equity of health services [19]. Internationally, models of school-based health clinics are thought to fill an important gap in health service provision, particularly for underserved populations, addressing availability, accessibility and equity [20-23]. Health service acceptability by youth may be further understood as perceived confidentiality, embarrassment in disclosing health concerns, stigma from peers, knowledge of the service, and trust in the health professional [24], all of which were also of importance to students in the current study.

Low levels of youth help-seeking, especially for issues of mental and sexual health, is a global phenomenon [18]. An international review of adolescents’ help-seeking behaviours indicated that 70% to 90% of young people will attend a health provider for primary care, but considerable unmet need was identified for drug and alcohol use, sexual health and mental health [25]. The current study found that the SYHN Program is providing accessible and acceptable primary health care addressing these areas of importance to youth health. Almost 80% of contacts with the nurses were for mental health, sexual health or drug and alcohol issues. Similar to other Australian programs [5,9,26], the evidence also demonstrates that the SYHNs are able to successfully connect with, and provide access to, external services for high-risk groups.

Around three quarters of consultations across the two years involved girls, a finding consistent with other research on male adolescents seeking non-injury or illness health care [27]. Factors identified by other researchers that may be considered for future development in the SYHN Program to improve engagement with boys include student choice over the sex of the health provider [3], longer hours of access [28], and parental, particularly maternal, support [29].

### Health promotion model

More than three quarters of students who participated in the survey indicated that they were aware of the nurse in their school. This was primarily attributable to large scale education activities such as the nurse presenting at assemblies or providing talks as part of the curriculum. This represents greater awareness than reported in other studies such as the British Youth Council [3]. However, this finding should be interpreted with some caution as some schools purposively sampled grades with higher rates of individual and class-based contact with the nurse to maximise the opportunity for feedback on the Program.

The health promotion approach is known to empower young people to make informed choices regarding health-related behaviours such as sexual health, smoking, alcohol and other drug use, and mental health [30-32]. This evaluation provides evidence that the SYHN Program is implementing a comprehensive approach to health promotion via contributions to all six components of a health promoting school: school health policies, school physical environment, school social environment, community involvement, curriculum to develop health skills and the provision of health services at the school with a health promotion focus [2].

### Effects on health and beyond

Qualitative and quantitative evidence from this study suggests that the SYHN Program is succeeding in supporting the objectives of the Health Promoting Schools framework [33]. Students who had accessed the Program were very satisfied with the experience, and almost half reported an improvement in their health. Further, school staff observed that the Program was having a positive impact beyond health such as improved school attendance and educational outcomes for some high-risk students. This supports the Royal College of Nursing statement that the effects of school nurses range further than health, potentially impacting on social and life choices and breaking detrimental intergenerational cycles [34] and is consistent with strong evidence that Health Promoting Schools are effective in improving the health and wellbeing of students [35].

### Scope of practice

Nurses, school staff and students experienced some initial uncertainty about the scope and nature of the role, similarly reported for other programs [9]. Over the course of the pilot, the nurses in the SYHN Program have become a respected source of health information in the schools, consulted on curriculum development and contributing to whole-of-school health activities. Some study participants, particularly from other youth health services, suggested that the scope of activities for the SYHNs could in fact be expanded to fully utilise their clinical skills, but it is not clear whether this would shift the health promotion, support and referral emphasis of the Program to greater health service provision.

### Time allocation

Consistent with reports on other school nursing programs, [5,9,26] the two day allocation of nurse time at each school was reported as a barrier across the RE-AIM dimensions.

Fulfilling the potential of the SYHN Program to provide both whole of school health promotion as well as primary health care access will be facilitated by a flexible approach to nurse time allocation according to need. There is good evidence that having a nurse in the school setting significantly reduces the time burden of health issues on other staff and is cost-effective [36]. Improving provision of other resources such as appropriate room space, support to access school IT systems, and consistent provision of health promotion tools such as posters would also be beneficial [34,37,38].

The need is likely higher in areas of socioeconomic disadvantage and particular attention may need to be paid to the nurses’ capacity in these schools. The two day allocation may be too simplistic to address underlying issues of equity. As noted in a review of the Victorian Program [26], an allocation model that accounts for school size and uses indices of relative risk may assist in both supporting school need and reducing nurse burnout.

### Integration into school

The SYHN model allows nurses to become an integrated part of the school community, contributing to broader social and educational as well as health outcomes. Successful integration requires considerable cross-sector collaboration during planning and flexibility in implementation. Evidence from other studies confirms that collaboration and mutual respect lead to greatest role satisfaction for nurses [5,26,38-40].

Consistent with reports from other Australian programs based on this model, the primary threat to integration was tension caused by the issue of confidentiality which differed fundamentally between nurses (following their policy on health professional confidentiality) and educators (following education policy on duty-of-care) [5,9,26]. This tension can create teamwork challenges and warrants careful and ongoing consideration and discussion when such programs are implemented.

### Limitations of this evaluation

As this evaluation was retrospective, there were no measures of change in youth health outcomes. The distribution of the student survey was carried out by school principals and was highly variable across schools, resulting in response rates ranging from less than 1% to 18% of the school enrolments for grades 7–10, with 7% of the overall enrolments for the schools completing the questionnaire. The ethical requirement for active written consent from both student and parent likely affected the representativeness of the sample. The short time period for the evaluation coupled with the numerous approvals required to conduct the study restricted the time available for data collection and the scope to employ alternative methods to improve survey response. However, student respondents did include diversity of age, gender and school grade. The low parent response to the survey was not sufficient to evaluate parent perceptions of the Program.

## Conclusion

This evaluation found that the SYHN Program is delivering accessible and acceptable primary health care, focused on health promotion, and delivered both individually and through group education. The Program implementation is consistent with the SYHN aims based on the Health Promoting Schools framework.

Whilst the evaluation was somewhat limited by the lack of prospective health outcome measures built into the Program, the evidence demonstrates that the SYHN Program is successfully connecting youth, a known hard-to-reach population, with a trusted source of health information and referral. Some work remains to be done on points of occasional tension such as confidentiality and scope of practice, but overall the SYHN model offers considerable opportunity for primary health care provision for adolescents in the school setting. The evidence gathered in this evaluation supports the expansion of the program as part of the ACT Government commitment to the health and wellbeing of young people.

## Additional file

Additional file 1:
**Indicative interview questions.**

## Abbreviations

ACT: Australian Capital Territory
ATSI: Aboriginal and Torres Strait Islander
CALD: Culturally and linguistically diverse
MOU: Memorandum of Understanding
RE-AIM: Reach, Effectiveness, Adoption, Implementation, Maintenance
RN: Registered Nurse
SYHN: School Youth Health Nurse
WHO: World Health Organization
