# Supplementary material for: Health promotion in schools: a multi-method evaluation of an Australian School Youth Health Nurse Program
Source: BMC Nurs. 2015 Apr 22;14:21. doi: 10.1186/s12912-015-0071-0 (PMC4416321; doi:10.1186/s12912-015-0071-0)
Supplement: Additional file 1: — Indicative interview questions. [file 12912_2015_71_MOESM1_ESM.pdf]

Question area	RE-AIM dimension	Participants
Strategies used to promote the program	Reach, Adoption	Nurses<br>School staff<br>External stakeholders
Development of role	Reach, Implementation	Nurse
Impact on youth health and wellbeing	Effectiveness	School staff<br>Government stakeholders
Impact on external organisations	Effectiveness	External stakeholders
School staff members involved with program	Adoption	Nurses<br>School staff
External organisations supporting program	Adoption	Nurses<br>External stakeholders
Barriers to reach and adoption	Reach, Adoption	Nurses<br>School staff<br>External stakeholders
Enablers to reach and adoption	Reach, Adoption	Nurses<br>School staff<br>External stakeholders
Program development	Implementation	Government stakeholders
Program implementation according to guidelines	Implementation	Nurses<br>Government stakeholders
Balance between individual, group and whole school activities	Implementation, Maintenance	Nurses<br>School staff
Problems with implementation	Implementation	Nurses<br>Government stakeholders
Areas that need improvement to increase effectiveness	Implementation	Nurses<br>School staff<br>External stakeholders
Areas that work well	Effectiveness, Implementation	Nurses<br>School staff<br>External stakeholders
Integration into schools	Maintenance	School staff<br>Government stakeholders
Processes for quality improvement	Maintenance	Nurses

and sustainability		Government stakeholders
Opportunities for further education, mentoring and supervision	Maintenance	Nurses
Satisfaction with role (interview)	Maintenance	Nurses
